# Supplementary material for: Multicenter Observational Study on Metastatic Non-Small Cell Lung Cancer Harboring BRAF Mutations: Focus on Clinical Characteristics and Treatment Outcome of V600E and Non-V600E Subgroups
Source: Cancers (Basel). 2022 Apr 16;14(8):2019. doi: 10.3390/cancers14082019 (PMC9031288; doi:10.3390/cancers14082019)
Supplement: Supplementary file 1 [file cancers-14-02019-s001.zip › cancers-1684318-supplementary.pdf]

Supplementary Table S1. Patient Population Characteristics of the overall population and according to BRAF mutational status. SCC: Squamous Cell Carcinoma; ADC: Adenocarci-noma; NSCLC: Non-Small Cell Lung Cancer; NOS: Not Otherwise Specified, ECOG: Eastern Cooperative Group; TPS: Tumor Proportional Score.

**Patient characteristics**		**Overall (n = 44)**	***BRAF* V600E (n = 23)**	***BRAF* non-V600E (n = 21)**	**Adjusted *P*-value***
**Age, years (Median, range)**		67 (47-84)	79 (47-81)	65 (49-84)	0.808
		**n (%)**			
**Histotype**	**SCC**	1 (2)	1 (4)	0 (0)	**0.144**
	**ADC**	36 (82)	17 (74)	19 (91)	
	**NSCLC NOS**	7 (16)	5 (22)	2 (9)	
**Sex**	**Male**	28 (64)	16 (70)	12 (57)	1.000
	**Female**	16 (36)	7 (30)	9 (43)	
**Smoking status***	**Smokers**	9 (24)	3 (14)	6 (37)	**0.192**
	**Ex-Smokers**	23 (60)	13 (59)	10 (63)	
	**Non Smokers**	6 (16)	6 (27)	0 (0)	
**ECOG PS**	**0-1**	39 (89)	21 (91)	18 (86)	1.000
	**2**	5 (11)	2 (9)	3 (14)	
**Stage of the disease**	**IIIB**	4 (9)	2 (9)	2 (9)	1.000
	**IV**	40 (91)	21 (91)	19 (91)	
**Number of metastatic sites**	**< 3**	17 (39)	10 (43)	7 (33)	1.000
	**≥ 3**	27 (61)	13 (57)	14 (67)	
**Metastatic Involvement**	**Bone**	14 (32)	7 (30)	7 (33)	1.000
	**Brain**	8 (18)	3 (13)	5 (24)	1.000
	**Liver**	7 (16)	5 (22)	2 (9)	1.000
	**Lung**	20 (45)	11 (48)	9 (43)	1.000
	**Lymph nodes**	32 (73)	15 (65)	17 (81)	1.000
	**Adrenal gland**	5 (11)	4 (17)	1 (5)	1.000
	**Pleura**	14 (32)	9 (39)	5 (24)	1.000
**First-line Treatment**	**Chemotherapy**	30 (68)	14 (61)	16 (76)	0.486
	**Immunotherapy**	9 (21)	4 (17)	5 (24)	
	**Targeted therapy** §	5 (11)	5 (22)	0 (0)	
**PD-L1 status**	**TPS ≥ 50%**	16 (37)	30 (23)	47 (28)	1.000
	**1% ≤ TPS < 50 %**	7 (16)	42 (32)	59 (35)	

**TPS < 1%**	8 (18)	51 (39)	48 (29)
**Unknown**	13 (29)	7 (6)	13 (8)

* In 6 patients the data was not available

§ 4 patients were treated with Dabrafenib + Trametinib, 1 patient with Encorafenib + Binimetinib

*P values are referred to the comparison between BRAF V600E and non-V600E groups (Fisher's exact test for categorical variables; Mann Whitney test for continuous variables). *Adjusted P values following Bonferroni correction.*

Please, note that when the product of the P values and the number of comparisons exceeds 1, the Bonferroni-corrected P value will be 1.000.

Supplementary Table S2. Explanatory prognostic factors in Cox proportional hazard models: uni- and multivariate analysis illustrating the impact on OS and PFS of clinico-pathological characteristics.

**OS, Univariate analysis**[a]	**Overall**				**BRAF V600E**				**BRAF non-V600E**			
	**HR**	**CI (95%)**	χ^2	***P* value***	**HR**	**CI (95%)**	χ^2	***P* value***	**HR**	**CI (95%)**	χ^2	***P* value***
**Age**	1.05	1.01-1.10	5.73	**0.023**	1.02	0.96-1.08	0.54	0.477	1.07	1.01-1.15	4.75	**0.030**
**Histotype**	1.18	0.78-1.79	0.58	1.000	2.16	1.04-4.49	3.97	**0. 120**	0.88	0.45-1.75	0.14	0.725
**Sex**	0.51	0.22-1.21	2.40	0.110	0.48	1.14-1.70	1.49	0.255	0.61	1.18-2.01	0.69	0.414
**Smoking status**	1.06	0.53-2.10	0.03	0.869	1.32	0.54-3.23	0.38	0.539	1.24	0.29-5.29	0.08	0.772
**ECOG PS**	4.17	1.38-12.63	4.77	**0.011**	1.39	0.18-11.04	0.09	0.754	18.6	3.0-75.68	8.89	**0.002**
**N of metastatic sites**	0.98	0.46-2.08	0.03	0.956	1.18	0.43-3.30	0.74	1.185	0.88	0.27-2.94	0.04	0.841
**Bone metastasis**	1.48	0.64-3.39	0.82	0.356	1.91	0.55-6.63	0.97	0.307	1.18	0.37-3.78	0.08	0.775
**Brain metastasis**	0.94	0.35-2.49	0.02	0.901	4.67	1.14-19.07	3.71	**0.032**	0.52	0.11-2.43	0.79	0.406
**Liver metastasis**	2.26	0.87-5.91	2.45	0.095	2.18	0.62-7.60	1.37	0.222	2.68	0.54-13.1	1.21	0.229
**Lymphnodes metastasis**	1.19	0.53-2.67	0.18	0.668	2.09	0.70-6.25	1.88	0.184	0.58	1.15-2.14	0.62	0.410
**Adrenal metastasis**	1.48	0.43-5.08	0.36	0.531	2.89	0.71-11.79	1.91	0.139	0.04	0.03-9.11	1.64	0.537
**I line treatment**	1.13	0.82-1.55	0.49	1.000	1.14	0.80-1.61	0.48	1.000	1.38	0.37-1.12	0.22	1.000
**PD-L1 status, TPS**	1.27	0.72-2.23	0.71	1.000	1.16	0.48-2.79	0.11	1.000	1.29	0.57-2.96	0.38	1.000

**PFS, Univariate analysis**[a]	**Overall**				**BRAF V600E**				**BRAF non-V600E**			
	**HR**	**CI (95%)**	χ^2	***P* value***	**HR**	**CI (95%)**	χ^2	***P* value***	**HR**	**CI (95%)**	χ^2	***P* value***

**Age**	0.98	0.94-1.02	1.25	0.257	0.98	0.93-1.03	0.61	0.430	0.98	0.91-1.06	0.18	0.673
**Histotype**	0.83	0.52-1.34	0.65	1.000	1.11	0.52-2.38	0.08	1.000	0.76	0.38-1.51	0.75	1.000
**Sex**	0.91	0.42-1.97	0.06	0.810	1.47	0.48-4.54	0.43	0.504	0.19	0.05-0.79	5.97	**0.022**
**Smoking status**	1.44	0.77-2.68	1.32	0.762	0.89	0.37-2.17	0.06	1.000	1.61	0.54-4.82	0.71	1.000
**ECOG PS**	3.56	0.42-29.84	1.02	0.242	8.64	0.78-15.8	2.21	0.079	0.04	0.01-7.89	0.35	0.776
**N of metastatic sites**	1.39	0.62-3.11	0.66	0.422	1.53	0.49-4.72	0.57	0.455	0.87	0.27-2.81	0.05	0.813
**Bone metastasis**	2.60	1.16-5.81	5.09	**0.020**	2.22	0.64-7.69	1.45	0.209	2.60	0.89-7.56	2.99	0.079
**Brain metastasis**	2.31	0.84-6.37	2.28	0.103	2.28	0.47-11.1	0.89	0.306	3.63	0.79-16.5	2.48	0.096
**Liver metastasis**	1.19	0.47-2.99	0.13	0.714	1.75	0.51-6.03	0.73	0.378	0.99	0.21-4.54	0.01	0.986
**Lymphnodes metastasis**	0.96	0.43-2.15	0.01	0.925	0.99	0.33-2.99	0.01	0.993	2.17	0.04-2.78	4.35	**0.023**
**Adrenal metastasis**	1.60	0.54-4.75	0.65	0.395	0.48	0.61-9.01	1.43	0.204	1.37	0.17-11.09	0.08	0.766
**I line treatment**	0.79	0.51-1.21	1.48	0.834	0.87	0.56-1.33	0.48	1.000	0.63	0.14-2.82	0.41	1.000
**PD-L1 status, TPS**	0.72	0.40-1.28	1.21	0.801	0.69	0.26-1.83	0.53	1.000	0.96	0.44-2.09	0.01	1.000

PFS: progression free survival; OS: overall survival; Age (continue variable), Histotype (SCC=0, ADC=1, NOS=3), Smoking status (negative smoking history=0, positive smoking history=1), Sex (Male=0, Female=1), Eastern Cooperative Oncology Group performance status (ECOG PS, 0-1 vs 2), number of metastatic sites (< 3 = 0, ≥ 3 = 1), bone/brain/liver/lymphnode/adrenal gland metastasis (absence=0, presence=1), first line treatment (chemotherapy=0, immunotherapy=1, chemo-immunotherapy=2), PD-L1 status (TPS < 1% = 0, 1-49% = 1, ≥ 50% = 2);

Statistical results with **P** < 0.05 are bolded.

a Univariate analysis is carried out without any adjustment.

*Adjusted P values following Bonferroni correction.

Please, note that when the product of the P values and the number of comparisons exceeds 1, the Bonferroni-corrected P value results 1.000.
